# Cardiac output Optimisation following Liver Transplant (COLT) trial: study protocol for a feasibility  randomised controlled trial

**DOI:** 10.1186/s13063-018-2488-8

**Published:** 2018-03-07

**Authors:** Farid Froghi, Rahul Koti, Kurinchi Gurusamy, Susan Mallett, Douglas Thorburn, Linda Selves, Sarah James, Jeshika Singh, Manuel Pinto, Christine Eastgate, Margaret McNeil, Helder Filipe, Fatima Jichi, Nick Schofield, Daniel Martin, Brian Davidson

**Affiliations:** 1 Division of Surgery and Interventional Science, University College London, London, UK; 2 Critical Care Unit, Royal Free Hospital, London, NW3 2QG UK; 3 Royal Free Perioperative Research Group (RoFPoR), Royal Free Hospital, London, UK; 4 Institute for Liver and Digestive Health, University College London, London, UK; 5 Health Economic Research Group, Brunel University, London, UK; 6 Biostatistics Group, Joint Research Office, University College London, London, UK

**Keywords:** Fluid therapy, Liver transplantation, Cardiac output, Perioperative care

## Abstract

**Background:**

Patients with liver cirrhosis undergoing liver transplantation have a hyperdynamic circulation which persists into the early postoperative period making accurate assessment of fluid requirements challenging. Goal-directed fluid therapy (GDFT) has been shown to reduce morbidity and mortality in a number of surgery settings. The impact of GDFT in patients undergoing liver transplantation is unknown. A feasibility trial was designed to determine patient and clinician support for recruitment into a randomised controlled trial of GDFT following liver transplantation, adherence to a GDFT protocol, participant withdrawal, and to determine appropriate endpoints for a subsequent larger trial to evaluate the efficacy of GDFT in patients undergoing liver transplantation.

**Methods:**

The Cardiac output Optimisation following Liver Transplant (COLT) trial is designed as a prospective, single-centre, randomised controlled study to assess the feasibility and safety of GDFT in liver transplantation for patients with cirrhosis. Consenting adults (aged between 18 and 80 years) with biopsy-proven liver cirrhosis who have been selected to undergo a first liver transplantation will be included in the trial and randomised into GDFT or standard care starting immediately after surgery and continuing for the first 12 h thereafter. Both groups will have cardiac output and stroke volume monitored using the FloTrac (EV1000) device. The intervention will consist of a protocolised GDFT approach to patient management, using stroke volume optimisation. The control group will receive standard care, without stroke volume and cardiac output measurement. After 12 h the patient’s fluid management will revert to standard of care. The primary endpoint of this study is feasibility. Secondary endpoints will include a safety assessment of the intervention, graft and patient survival, liver function, postoperative complications graded by Clavien-Dindo criteria, length of intensive care and hospital stay and quality of life across the intervention and control groups.

**Discussion:**

There is a growing body of evidence that the use of perioperative GDFT in surgical patients can improve outcomes; however, signals of harm have also been detected. Patients with liver cirrhosis undergoing liver transplantation have markedly different cardiovascular physiology than general surgical patients. If GDFT is proven to be feasible and safe in this patient group, then a multicentre trial to demonstrate efficacy and cost-effectiveness will be required.

**Trial registration:**

International Standard Randomised Controlled Trial Registry, ID: ISRCTN10329248. Registered on 4 April 2016.

**Electronic supplementary material:**

The online version of this article (10.1186/s13063-018-2488-8) contains supplementary material, which is available to authorized users.

## Background

Goal-directed fluid therapy (GDFT) aims to deliver the correct amount of intravenously administered (IV) fluids to patients at the correct time, thus avoiding the well-documented detrimental consequences of either inadequate or excessive IV fluids. Several clinical trials in surgical patients have shown the benefit from using haemodynamic (cardiac output) monitoring coupled with a validated protocol for IV fluids in reducing postoperative morbidity and hospital length of stay; however, signals of harm have been detected [1, 2]. Hence, fluid therapy strategies are increasingly refined for each clinical scenario taking into account the disease processes involved. This goal-directed approach to perioperative fluid management has shown a reduction in intensive care unit (ICU) utilisation and hospital length of stay in the general surgical population [3]. Over the last two decades, the development of numerous non-invasive haemodynamic monitoring devices has allowed assessment of cardiac output (and stroke volume (SV)) without the need for invasive techniques such as pulmonary artery catheterisation [4]. The academic literature supporting their use in the perioperative period is rapidly expanding and a recent National Institute of Health and Care Excellence (NICE) guideline recommended that a cardiac output monitor should be used in all major surgery [5]. This document also highlighted the significant cost savings when the GDFT approach to perioperative care is used, through the avoidance of complications and excessive hospital length of stay. A single, major, postoperative complication costs the NHS approximately £10,000 [6].

Patients with cirrhosis of the liver undergoing liver transplantation pose a unique challenge in that they have the altered haemodynamics associated with end-stage cirrhosis compounded by the significant blood loss of transplantation and the systemic changes associated with ischaemia-reperfusion graft injury [7–9]. Therefore, data supporting the use of GDFT following other forms of high-risk surgery may not be applicable to this patient cohort. It is conceivable that GDFT could do harm in this complex patient population, as shown in other settings [10]. In severe acute pancreatitis, early high-volume fluid administration, as was routinely recommended in clinical practise guidelines, has been shown to increase mortality as well as complications [11]. Cirrhosis is associated with a variety of circulatory changes including portal hypertension, venous shunting, peripheral venous dilatation and a hyperdynamic circulation with an elevated cardiac output that persists into the early post-transplant period [12–14]. Measurements of central venous and pulmonary capillary wedge-pressures have traditionally been used to try and identify hypovolaemia post liver transplantation but both values are known to be limited in their ability to do this [15]. Less invasive pulse-contour analysis devices, such as the FloTrac (EV1000) device, have been used for cardiac output measurement with reasonable accuracy in patients undergoing liver transplantation [16, 17]. Whilst there are many donor and recipient factors that contribute to graft and patient survival [18, 19], optimal perfusion of the graft is the final common pathway for many of the factors influencing outcome and optimising hepatic blood flow following liver transplant has been shown to result in improved graft function [20].

A feasibility trial was designed to determine patient and clinician support for recruitment into a randomised controlled trial (RCT) of GDFT in liver transplantation, adherence to a GDFT protocol, participant withdrawal and to determine appropriate endpoints for a subsequent RCT to evaluate the efficacy and costs-effectiveness of GDT in patients undergoing liver transplantation.

## Methods

### Study design and setting

**Fig. 1:**
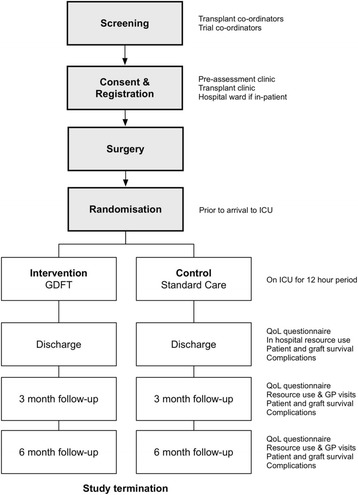
Trial flow diagram

**Fig. 2:**
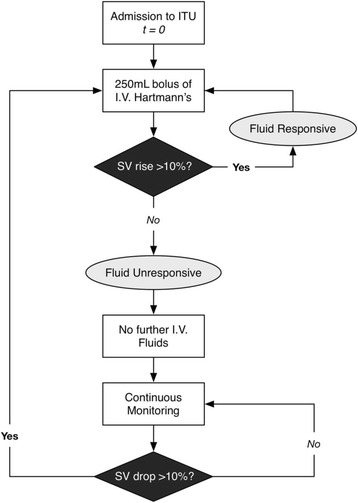
Schedule of enrolment, interventions and assessments

The COLT trial has been designed using the Standard Protocol Items: Recommendations for Interventional Trials (SPIRIT) guidelines (see Additional file 1) as a prospective, single-centre, randomised controlled feasibility trial [21]. The initial feasibility trial is to take place at the Royal Free London NHS Foundation Hospital Trust (RFLH), one of the eight designated liver transplant centres in UK with over 100 orthotopic liver transplants per year with a view to conducting a subsequent multicentre, cost-effectiveness trial. The trial is sponsored by University College London (UCL) and is funded by a National Institute for Health Research (NIHR) Research for Patient Benefit (RfPB grant no. PB-PG-0214-33043). A trial outline is illustrated in Fig. 1 and the schedule of enrolment, interventions and assessments is shown in Fig. 2.

### Ethical approval

The study was reviewed and approved by University College London Bloomsbury Research Ethics Committee (Ref: 180463) and has been registered at International Standard Randomised Controlled Trial Registry (ISRCTN10329248 10.1186/ISRCTN10329248) on 4 April 2016.

### Participants

Consenting adult patients (aged between 18 and 80 years) with liver cirrhosis selected to undergo liver transplantation will be included in the trial. The exclusion criteria will be those with non-cirrhotic liver disease, pregnancy, age less than 18 years or over 80 years, body weight less than 40 kg, re-transplantation for primary graft non-function, fulminant hepatic failure, emergency surgery, known learning disabilities or previously lacking capacity to consent for themselves or with previous episodes of encephalopathy, prisoners, patients already enrolled in an interventional study and refusal or inability to consent.

### Recruitment

Patients are screened in the transplant assessment clinic and are provided with the trial Patient Information Sheet (PIS) directly or by post. The trial is then discussed with a team member in depth at the time of the subsequent short-stay inpatient assessment. Informed consent is then obtained by a member of the research team and a baseline quality of life (QoL) assessment is performed.

### Power calculation

This is a feasibility study and a power calculation is, therefore, not required. A total of 50 patients will be recruited with 25 in the GDFT and 25 in standard care treatment arms.

### Randomisation

We will use a commercially available clinical randomisation service (http://www.sealedenvelope.com) immediately after liver transplantation at the time of admission to the intensive care unit (ICU). Patients will be randomised to one of the two groups (GDFT versus standard care) using stratified random permuted blocks of varying block sizes to ensure similar numbers in the groups whilst maintaining blinding and concealed allocation. Randomisation will be stratified by donor type (Deceased after Cardiac Death (DCD) donor and Deceased after Brain Death (DBD) donor) to achieve approximate balance in this characteristic. Patients will be randomised after surgery prior to arriving in ICU to either the GDFT group or the standard care group on a 1:1 basis.

### Intervention

**Fig. 3:**
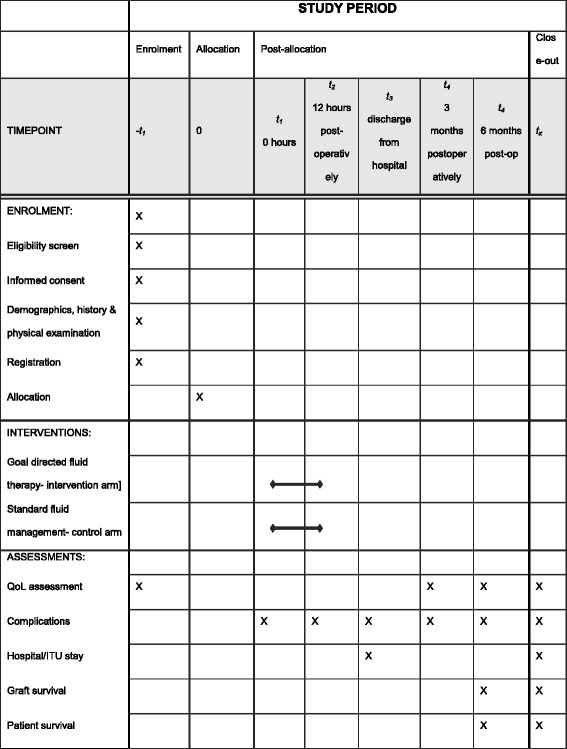
Goal-directed fluid therapy (intervention) protocol

The trial intervention period will commence when the patient arrives on the ICU following transplant surgery and will continue for 12 h postoperatively. In both study groups (GDFT and control), a FloTrac sensor (EV1000 Clinical Platform, Edwards Life Sciences, Irvine, CA, USA) will be used to measure SV and cardiac output. In the GDFT group only, this information will be used to guide IV fluid therapy according to the study protocol (Fig. 3). The intervention consists of an initial bolus infusion of 250 mL Hartmann’s on arrival to ICU. If this leads to a > 10% increase in SV the patient is deemed to be fluid responsive and the bolus is repeated until no further SV response is achieved (i.e. euvolaemia). SV is continuously monitored and the bolus is repeated if the SV drops by 10%. There are no extra maintenance fluids in those who are not fluid responsive.

The FloTrac (EV1000) cardiac output monitor is a non-invasive, pulse-wave-contour-analysis device that estimates cardiac output and SV from an arterial line trace. We will endeavour to use the radial artery in all cases to standardise the approach. This device has been shown to reliably track other accepted methods of monitoring cardiovascular haemodynamics [4].

The control group will receive standard care as per ICU clinician choice and judgement. This will of course be varied and not normally based on cardiac output measures.

### Blinding

A COLT trial research nurse will collect SV and cardiac output data in the control group but these values will not be available to the ICU team managing the post-transplant fluid administration. The trial nurse and clinical team will be aware of the participant’s group allocation. The anaesthetic team involved in the liver transplant procedure will be blinded to the postoperative randomisation as will the hepatology and surgical teams involved in the postoperative management. Data will be collected on complications and outcomes and presented to the Data Monitoring Committee (DMC) as groups A and B with the identification of the groups remaining blinded.

During the 12-h study period all other medical care will be similar in the two groups and once the trial period is complete, patients will return to standard medical care with fluid administration as guided by the patient’s clinical team. The research nurses conducting this trial will all be experienced intensive care nurses, trained to use the EV1000 and will follow the GDFT protocol.

### Outcome measures

The primary outcome of this study is feasibility. A recruitment rate of greater than 40% of patients fulfilling the criteria for this study will be deemed successful. We shall assess patient recruitment, clinician support for the trial, completion rate of the intervention and reasons for participant withdrawal from the trial. Secondary outcomes will explore the safety of the intervention, and to assess endpoints in order to determine the most appropriate endpoints for a future trial of efficacy, and to facilitate sample size calculation. All transplant-related and other complications will be recorded throughout the inpatient stay as well as at 3 and 6 months’ follow-up. To monitor safety, complications likely to be related to the intervention will be reported and reviewed as a potential serious adverse event (SAE). The safety profile and complication rates in both arms (blinded) will be reviewed regularly by an external Data Monitoring Committee (DMC). Most studies evaluating the benefit of GDFT choose length of hospital stay or incidence of postoperative complications as endpoints. In the high-risk scenario of liver transplantation, long-term graft (transplanted liver) function and patient survival may be more appropriate endpoints. However, as the outcomes of liver transplantation continually improve, these endpoints may not be achievable and other clinically relevant endpoints, such as number and severity of complications or health-related quality of life (QoL), may be more appropriate.

A recent liver transplant patient and public involvement (PPI) forum held at the Royal Free Hospital sought the views of liver transplantation patients regarding the most important outcome measures in liver transplant research. Health-related QoL was deemed to be the primary endpoint which was most useful in liver transplant trials. A composite measure of QoL and survival called quality-adjusted life years (QALYs) will be calculated in the study. QoL will be measured using the EuroQol five dimensions, five levels (EQ-5D-5 L) questionnaire which is recommended by NICE to inform cost-utility analyses of healthcare interventions [22].

Resource-use data will be collected during the trial which will include core cost components during the inpatient stay as well as costs associated with the conduct of GDFT and the treatment of postoperative complications. Resources will be costed using national data sources where available, supported by locally held costing data and will be used to estimate differences in costs between the treatment arms. The difference in total costs and QALYs across the control and GDFT groups will be assessed. Given the small sample size, an incremental cost per QALY will be not be estimated.

A recruitment rate of greater than 40% will be the criteria to progress to a subsequent efficacy and cost-effectiveness trial. We will not proceed to a subsequent trial if there is a 10% or greater complication rate in the intervention group than those seen in the control group.

### Data collection and analysis

Each participant will be given a unique trial Participant Identification Number (PIN). The health-related QoL (EQ-5D-5 L questionnaire) will be collected at the baseline, hospital discharge and at 3 and 6 months’ follow-up. Utility value will be estimated from the EQ-5D-5 L data at different time points using the crosswalk algorithm developed by van Hout [23], which maps EQ-5D-5 L responses to an EQ-5D-3 L UK value set, as per the new NICE position statement and NICE reference case [23–25]. Continuous haemodynamic data from the FloTrac device will be collected during the study period for both arms of the study as well as intraoperative and postoperative IV fluid infusions (including IV fluids, inotropes, vasopressors and transfusions of blood and blood products). The volumes and timings of IV fluid boluses according to the SV changes will also be recorded in the GDFT arm. Complications will be recorded and graded according to Clavien-Dindo classification along with treatment costs [26].

The two treatment groups will be compared to ensure that they have similar baseline characteristics including cirrhosis aetiology using means and standard deviations or medians and inter-quartile ranges for continuous variables, as appropriate, and frequency counts and percentages for categorical variables. Information on donor characteristics (DBD or DCD), warm/cold ischaemia times, operative and reperfusion timing as well as technique of transplantation will also be collected and compared.

For feasibility outcomes, the proportion of patients who consent to be randomised will be presented with a 95% confidence interval. The proportion of patients withdrawn from GDFT will be presented, as well as the proportion of patients who deviate from the GDFT protocol for the 12-h intervention period.

All complications will be documented and graded by the Clavien-Dindo classification system. The mean difference in the proportion of people with a complication between the two groups will be calculated and the difference between those with a mild (Clavien-Dindo grades 1–3) versus severe (Clavien-Dindo grades 4 and 5) will be assessed. A 95% confidence interval will be presented. The median number of complications and the median grade of complications in each group will be presented. The median length of stay in ICU and in hospital will be presented for each group. The rate of readmission to ICU will also be presented for each group. Quality of life scores will be summarised for each group using mean profile plots over time. The mean difference in QoL scores between the two groups at 12 h will be presented with a 95% confidence. If the data is not normal, a median difference will be presented.

Kaplan-Meier plots will be presented to compare survival rates between the two groups.

All other secondary outcomes will be summarised for each group using mean profile plots over time. For binary secondary outcomes, the mean difference in proportion at 12 h between the two groups will be presented with 95% confidence intervals. For continuous secondary outcomes, mean/median differences at 12 h will be presented as appropriate, with 95% confidence intervals.

The results will inform us how sensitive the outcome measures are and, along with other information, will be presented to a focus group involving liver transplant patients and their carers, the COLT trial team, a group of transplant surgeons and physicians not involved with the trial and other major stakeholders including representatives of specialist societies. The results of the focus group will be used to determine the primary outcome of a subsequent multicentre RCT aimed at determining the efficacy of GDFT in liver transplantation and the costs involved. The results will also inform a sample size calculation to determine the number of patients required for the primary outcome chosen.

There is no planned interim analysis. However, the Data Monitoring Committee (DMC) will monitor the results after each 20 randomised patients. The patients will be grouped into a GDFT and a control group for the assessment of complications but the data will be presented to the DMC as two groups (A and B) without revealing which is the intervention (GDFT) and which is the sham control group. If the DMC feels that either group exceed the expected rate of complications or the anticipated morbidity and mortality, then the DMC may request unblinding of data. Potential complications that could be directly related to treatment are cardiac failure and thromboembolic manifestations. Serious adverse events (SAEs) are common following liver transplant. All events will be graded as to their possible or likely relationship to the initial fluid management and will be reported to the sponsor (Table 1).

**Table 1:** Common complications related to intravenously administered fluid therapy (modified from Hilton et al. 2008 [36])

Excess administration	Inadequate administration
• Peripheral oedema	• Tachycardia
• Hypertension	• Hypotension
• Respiratory failure	• Shock
• Cardiac failure	• Renal failure
• Poor wound healing	• Multiple organ failure
• Delayed bowel recovery	• Poor wound healing
• Electrolyte imbalance	• Electrolyte imbalance
• Coagulopathy	• Coagulopathy

### Dissemination

The results of this study will be presented to the British Transplant Society and prepared for publication in peer-reviewed journals with an interest in critical care and organ transplantation.

## Discussion

Every year 6000 to 7000 people in the UK, many of whom are in the prime of life, die from chronic liver disease which can be effectively treated with liver transplantation. However, with an increasing number of liver transplants being performed each year and a concomitant lack of suitable donor organs, transplant centres are using livers of marginal suitability. This is associated with an increased risk of postoperative complications along with reduced graft and patient survival. More than 800 liver transplants are performed per year in the UK, at a cost of approximately £80,000 per procedure. A large part of this substantial cost is due to the treatment of postoperative complications. There is consequently a major clinical and financial imperative in the NHS to reduce the complications post liver transplantation. The 1-year mortality following liver transplantation in the UK is 8.8% [27].

A recent analysis was carried out of complications following liver transplant at the Royal Free London NHS Foundation Trust. Of 551 patients who underwent liver transplantation from 1999 to 2008, 371 (67%) developed at least one significant postoperative complication. Those patients who developed a complication had a longer ICU stay (median of 3 versus 2 days) and longer hospital length of stay (median of 33 versus 21 days) than those who did not develop complications. Graft (transplanted liver) failure and mortality at 3 months postoperatively were also significantly higher in those with complications. In a more detailed recent audit from January 2011 to July 2012, data on 98 patients was examined. In this cohort 80% of patients developed at least one significant postoperative complication, with a median of two complications per patient. This apparent increase in postoperative complications is likely to reflect an increase in the number of marginal organs now used in liver transplantation and the increasing burden of comorbidities.

Rationalising postoperative fluid therapy to prevent iatrogenic harm and complications related to hyper- and hypovolaemia, such as renal failure and pulmonary oedema (Table 1), is an effective method of decreasing postoperative complications in surgical patients [28]. A goal-directed approach to fluid therapy using haemodynamic monitoring appears to have promising results in numerous randomised trials for elective and emergency surgical patients [29]. Patients with cirrhosis undergoing liver transplantation differ from the general surgical population in several ways which makes them a unique subset of patients.

Firstly, the disease process of cirrhosis entails a complex neuro-endocrine and hormonal activation, such as nitric oxide release and the renin-angiotensin system, creating a hyperdynamic circulation [30]. The haemodynamic physiology changes seen in cirrhosis (increased cardiac output, reduced systemic vascular resistance and effective central circulating blood volume) can last into the first 24-h period after liver transplantation [31]. This renders accurate assessment of intravascular volume status an exceptionally difficult task. The mechanism and timeline of alterations to these haemodynamic and neuro-endocrine changes after liver transplantation have not been adequately investigated. The COLT trial will provide an opportunity to study the response to fluid therapy in liver cirrhosis and investigate these underlying mechanisms.

Secondly, there are surgery-related factors influencing the patients’ haemodynamics after liver transplantation. Liver transplantation is high-risk surgery incurring huge surgical stress which is often prolonged in duration [32]. There are often pre-existing coagulation defects in cirrhotic patients with major blood loss during surgery requiring transfusion of blood and blood products and exacerbating the coagulopathy [8]. Major haemodynamic alterations are exacerbated by cross-clamping of the inferior vena cava during the transplant. Added to the surgical stress, there is the huge burden of ischaemia-reperfusion injury of the largest solid organ in the body which can affect vascular endothelial barrier function, activate inflammatory cascades and hence lead to undesired fluid shifts in the body and renal dysfunction [33]. These factors make it difficult to institute a fixed method of fluid assessment and replacement intraoperatively. Hence, in the COLT trial, the regimen of GDFT was instituted during the initial 12-h period postoperatively rather than during the operation.

There have been a number of retrospective analyses of the effect of fluid therapy on perioperative complications after liver transplantation. However, there have been no prospective randomised trials assessing GDFT post liver transplantation. Jiang et al. reported that the intra-operative fluid administration of > 100 mL/kg was associated with an increased time to extubation and length of ICU stay in a retrospective analysis of pulmonary complications post liver transplantation [34]. Another retrospective analysis of 62 patients showed that an intra-operative fluid infusion of > 9000 mL was a significant risk factor for developing postoperative respiratory complications [34]. Reydellet et al. reported, in a before and after retrospective study, that a GDFT approach for 2 days post liver transplantation results in decreased requirements for mechanical ventilation and duration of postoperative ileus [35]. The COLT trial is the first prospective RCT assessing GDFT in cirrhotic patients undergoing liver transplantation.

## Trial status

The COLT trial began recruitment in April 2017 and plans for completion of recruitment in approximately September 2017.

## Additional file

Additional file 1: SPIRIT 2013 Checklist: recommended items to address in a clinical trial protocol and related documents*. (DOC 120 kb)

## Abbreviations

CO: Cardiac output
COLT: Cardiac output Optimisation following Liver Transplantation
DBD: Donor after Brain Death
DCD: Donor after Cardiac Death
DMC: Data Monitoring Committee
GDFT: Goal-directed fluid therapy
ICU: Intensive care unit
PPI: Patient and public involvement
QALYs: Quality-adjusted life years
RCT: Randomised controlled trial
RFLH: Royal Free London Hospital
SAE: Serious adverse events
SV: Stroke volume
